# Switch-like genes populate cell communication pathways and are enriched for extracellular proteins

**DOI:** 10.1186/1471-2164-9-3

**Published:** 2008-01-04

**Authors:** Adam Ertel, Aydin Tozeren

**Affiliations:** 1 Center for Integrated Bioinformatics, School of Biomedical Engineering, Science and Health Systems, Drexel University, 3141 Chestnut Street, Philadelphia, PA 19104, USA

## Abstract

**Background:**

Recent studies have placed gene expression in the context of distribution profiles including housekeeping, graded, and bimodal (switch-like). Single-gene studies have shown bimodal expression results from healthy cell signaling and complex diseases such as cancer, however developing a comprehensive list of human bimodal genes has remained a major challenge due to inherent noise in human microarray data. This study presents a two-component mixture analysis of mouse gene expression data for genes on the Affymetrix MG-U74Av2 array for the detection and annotation of switch-like genes. Two-component normal mixtures were fit to the data to identify bimodal genes and their potential roles in cell signaling and disease progression.

**Results:**

Seventeen percent of the genes on the MG-U74Av2 array (1519 out of 9091) were identified as bimodal or switch-like. KEGG pathways significantly enriched for bimodal genes included ECM-receptor interaction, cell communication, and focal adhesion. Similarly, the GO biological process "cell adhesion" and cellular component "extracellular matrix" were significantly enriched. Switch-like genes were found to be associated with such diseases as congestive heart failure, Alzheimer's disease, arteriosclerosis, breast neoplasms, hypertension, myocardial infarction, obesity, rheumatoid arthritis, and type I and type II diabetes. In diabetes alone, over two hundred bimodal genes were in a different mode of expression compared to normal tissue.

**Conclusion:**

This research identified and annotated bimodal or switch-like genes in the mouse genome using a large collection of microarray data. Genes with bimodal expression were enriched within the cell membrane and extracellular environment. Hundreds of bimodal genes demonstrated alternate modes of expression in diabetic muscle, pancreas, liver, heart, and adipose tissue. Bimodal genes comprise a candidate set of biomarkers for a large number of disease states because their expressions are tightly regulated at the transcription level.

## Background

Gene expression microarrays have served as a useful tool for assaying large-scale similarities and differences among conditions including tissue types [1], stages of development [2,3], and disease states in humans [4,5] and model organisms [6]. Initial microarray classification studies such as those presented in [4,5] were able to characterize similarities and differences among samples based on mRNA expression level for large gene sets. More recent studies have made use of biological annotation, such as Gene Ontology (GO) or Kyoto Encyclopedia of Genes and Genomes (KEGG) pathways [7] to project changes in individual genes onto biological functions [8,9]. Existing biological annotation is also a useful supplement to machine learning techniques used for determining regulatory connections [10,11]. These techniques are sensitive to differential expression as well as small concerted changes in levels of gene expression, yet they may not adequately address changes with respect to the global behavior of gene expression – where transcript levels may either be tightly regulated within a narrow range, or fluctuate widely as a function of environmental cues or tissue specialization.

Efforts to explain biological functions associated with single genes or sets of related genes often focus on variations of gene expression across diverse tissue types. Identification of genes as tissue-selective and tissue-specific is useful for highlighting their biological function, as well as providing reference/context for disease states. Identification of tissue-specific and tissue-selective genes is commonly based on present/absent calls, requiring a global threshold [12-14]. Tissue-specific behavior has also been identified using statistical tests to compare sample distributions between tissue types [1,15,16]. Other approaches have used a numeric value representing the degree of tissue specificity within one tissue or tissue subset versus all others [17,18]. These studies are typically performed on a small number of samples within each tissue type; they nevertheless effectively describe genes with large variation between distinct tissues.

Efforts have been made to place gene expression in context of global behavior using descriptors such as breadth of gene expression [12] and distributions characteristics that represent ubiquitous, binary, or graded regulation [19-23]. Ubiquitously expressed "housekeeping" genes are defined as those highly expressed with little variation across conditions, and have been identified in humans using large-scale microarray studies [1,24]. While breadth of expression and housekeeping behavior have been established using genome-scale measurements, present descriptions of graded and binary genes have typically been produced using single-gene studies [19,20]. These studies have demonstrated that changes in gene expression levels can occur continuously or in a binary switch-like manner in response to extracellular changes. Binary modes of gene expression potentially correspond to those proteins with tight regulation at the transcript level. As such their identification is useful in the exposition of the multiple modes of gene expression regulation observed in eukaryotes.

In this study, we expand on the existing literature on gene expression profile distributions and determine a comprehensive list of bimodal genes along with their functional annotation. Our preliminary computations based on large collection of human microarray data indicated difficulties identifying profiles of bimodal expression due to a great degree of subject variability and noise. For this reason, the present study focuses on murine microarray data containing approximately 400 samples, all obtained using the Affymetrix MG-U74Av2 platform (Table 1) [3,6,25-39]. This new database allowed us to effectively apply a two-component mixture model to hundreds of data points for each gene and identify bimodal profiles. Moreover, bimodal genes with altered modes of expression were identified in microarray data for type I and type II diabetes (Table 2) [6,29,40,41]. Results point to important roles that bimodal (switch-like) genes play within the extracellular environment in health and disease. Bimodal genes, because they are tightly controlled around two distinct modes at the transcript level, serve as targets in drug development. Moreover, bimodal genes encoding for extracellular proteins may serve as biomarkers in targeted proteomic studies.

**Table 1:** Mouse gene expression datasets and tissue types composing our healthy dataset.

**Healthy Tissue Type**	**Accession No**.	**Source**	**Reference**	**Samples**	**Total**
Adipose	GSE480	GEO	N/A	4	6
	GSE2899	GEO	[6]	2	
Adrenal	GSE1674	GEO	[25]	6	6
Brain	GSE3327	GEO	[26]	87	89
	GSE480	GEO	N/A	2	
Colon	GSE2172	GEO	[27]	4	5
	E-MEXP-402	Array Express	N/A	1	
Epidermal	GSE1912	GEO	[28]	25	25
Heart	GSE77	GEO	N/A	30	38
	GSE4616	GEO	[29]	6	
	GSE480	GEO	N/A	2	
Kidney	E-MEXP-495	Array Express	[30]	3	3
Liver	E-MEXP-241	Array Express	[31]	6	8
	GSE2899	GEO	[6]	2	
Lung	GSE485	GEO	N/A	18	26
	GSE495	GEO	N/A	6	
	GSE480	GEO	N/A	2	
Mammary	GSE5831	GEO	N/A	9	15
	E-MEXP-892	Array Express	[32]	6	
Muscle	GSE469	GEO	[33]	54	64
	GSE1659	GEO	[29]	6	
	GSE2899	GEO	[6]	2	
	GSE480	GEO	N/A	2	
Ovary	GSE1359	GEO	[3]	10	10
Pancreas	GSE769	GEO	[34]	3	5
	GSE2899	GEO	[6]	2	
Peripheral Blood	GSE3039	GEO	[35]	12	12
Small Intestine	GSE765	GEO	N/A	3	3
Spleen	GSE5306	GEO	N/A	12	12
Stomach	E-MEXP-402	Array Express	N/A	1	1
Testis	GSE926	GEO	[36]	22	49
	GSE640	GEO	[37]	17	
	GSE1358	GEO	[3]	10	
Thymus	GSE2585	GEO	[38]	8	11
	GSE85	GEO	[39]	3	

All Healthy Tissues					388

**Table 2:** Mouse gene expression datasets and tissue types composing our diabetes dataset.

**Diabetic Tissue Type**	**Accession No**.	**Source**	**Reference**	**Samples**
Adipose (Type II)	GSE2899	GEO	[6]	2
Heart (Type I)	GSE4616	GEO	[29]	6
Liver (Type II)	GSE2899	GEO	[6]	2
Muscle (Type I)	GSE1659	GEO	[29]	6
Muscle (Type II)	GSE2899	GEO	[6]	2
Pancreas (Type I)	GSE1623	GEO	[40]	6
Pancreas (Type II)	GSE2899	GEO	[6]	2
Peripheral Blood (Type I)	GSE1419	GEO	[41]	22

## Results

### Identification of bimodal genes in the mouse genome

Our method identified 1519 bimodal genes out of the 9091 unique genes (17%) on the MG-U74Av2 array (see Additional file 1). The total number of bimodal genes was not sensitive to the p-value cutoff for bimodal versus skewed normal representations of the gene expression distribution within the ranges considered: the bimodal gene list increased by only three genes when the p-value was increased from 0.001 to 0.01. Similarly, gene expression outliers were not important contributors to the bimodal gene list. When we deleted the three largest gene expression values from the gene expression profile of each gene and ran our procedure for identification of bimodal genes, the resulting bimodal gene list turned out to be identical to our standard set of genes minus five genes. See Additional file 1 for a table that provides a comprehensive list of bimodal genes for the mouse genome. Columns of this table are composed of the following entities: Affymetrix probe ID, Entrez gene ID, gene symbol, human orthologs, log likelihood test statistic, estimated p-value, and maximum overlap *A*, representing the misclassification area between modes. Also listed for each gene in this table are parameters indicating the standardized distance between means *D*, the mixture parameter *π*, and the log RMA gene expression threshold value *X*_*T *_separating high and low expression modes. The information on this table constitutes a priori data needed to identify the high expression or low expression modes of each gene in any given sample. The table can be used to identify altered modes of expression in disease states provided that these genes preserve their bimodal expression patterns. The human orthologs of the bimodal mouse genes are listed in this table (Additional file 1) for reference and their bimodal behavior in humans would have to be verified in future studies.

### Tissue similarity based on common modes of expression within bimodal genes

**Figure 1:**
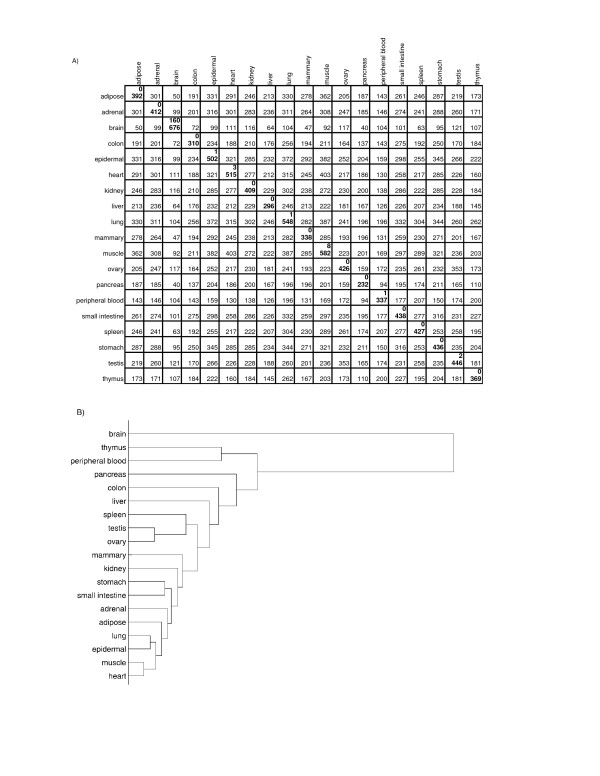
**Tissue selective genes common among nineteen tissue types**. A) The diagonal contains a top number corresponding to tissue-specific switch-like genes expressed in a given tissue and a bottom number identifying the total number of tissue-selective switch-like genes expressed in that tissue. B) A dendrogram representing tissue similarity based on tissue-selective expression of bimodal genes.

Next we considered similarity of the nineteen tissues for which we had extensive microarray data. As detailed in the methods section, we based our criteria of tissue similarity on the lists of tissue-selective bimodal genes in common within each unique pair of tissues. Figure 1 indicates that commonality in the set of tissue-selective bimodal genes is indicative of tissue similarity. The number of tissue-selective bimodal genes in the "high" mode for each tissue type is provided as the bottom number in the diagonal of Figure 1A, while the top number represents genes that may be considered tissue-specific; they are expressed in the "high" mode for that single tissue and the "low" mode for all others. The remaining matrix elements of Figure 1A are the number of bimodal genes in the "high" mode for both of the two tissue types designated in the row and column headings. We performed hierarchical clustering of the nineteen tissues based on sets of bimodal genes shared between them, to further demonstrate the role bimodal genes may play in tissue similarity. The dendrogram in Figure 1B was computed using hierarchical clustering with average linkage, using one over the number of bimodal genes shared between two tissues (from Figure 1A) as the distance metric. In several examples, tissues with similar function cluster together, such as stomach and small intestine, heart and skeletal muscle, thymus and peripheral blood, and the reproductive tissues ovary and testis, while brain clusters distinctly apart from all other tissues. Other groupings such as adipose, lung, adrenal, and epidermal tissue may occur because of signaling motifs shared among these tissue types. Our predications of tissue similarity are consistent with previous results that group human tissues by hierarchical clustering [1,12].

### Functional enrichment analysis indicates bimodal genes' involvement with the extracellular environment

**Figure 2:**
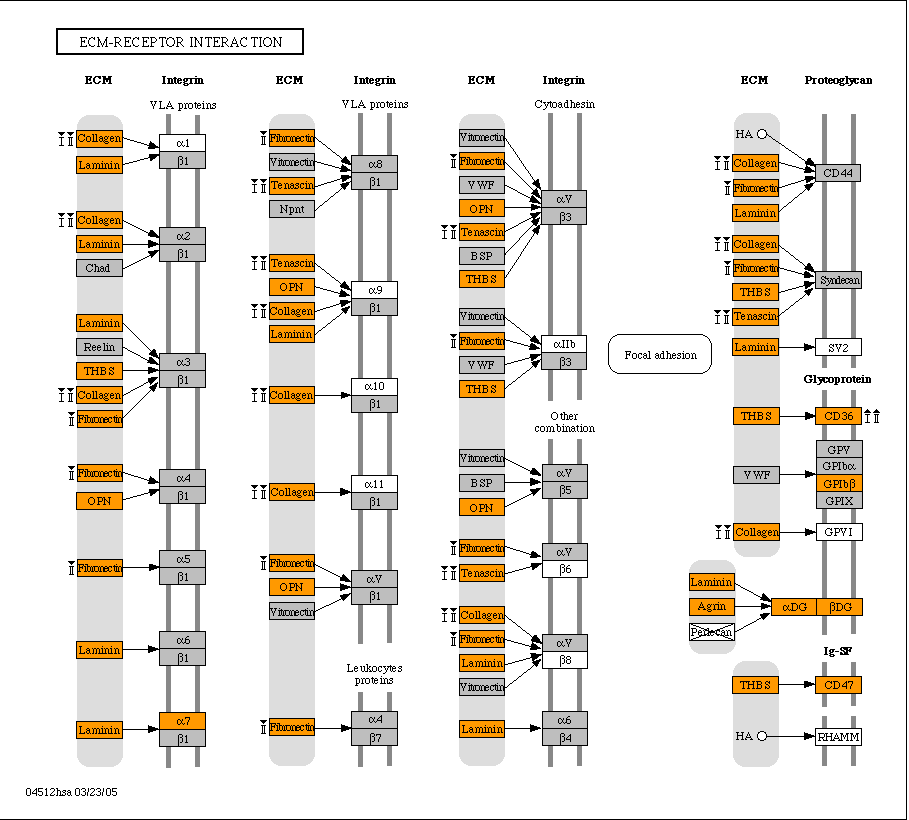
**Bimodal genes highlighted in KEGG ECM-receptor interaction diagram**. Nodes representing switch-like genes are colored orange; remaining genes on the MG-U74Av2 array are shown in grey, and white blocks designate genes not on the array. Genes not in the organism-specific pathway for mouse are crossed out. Genes identified as switching in skeletal muscle samples with diabetes type I and type II are labeled with "I" and "II", respectively, with arrows indicating the direction of change in the disease state (▲ is low-to-high; ▼ is high-to-low).

**Figure 3:**
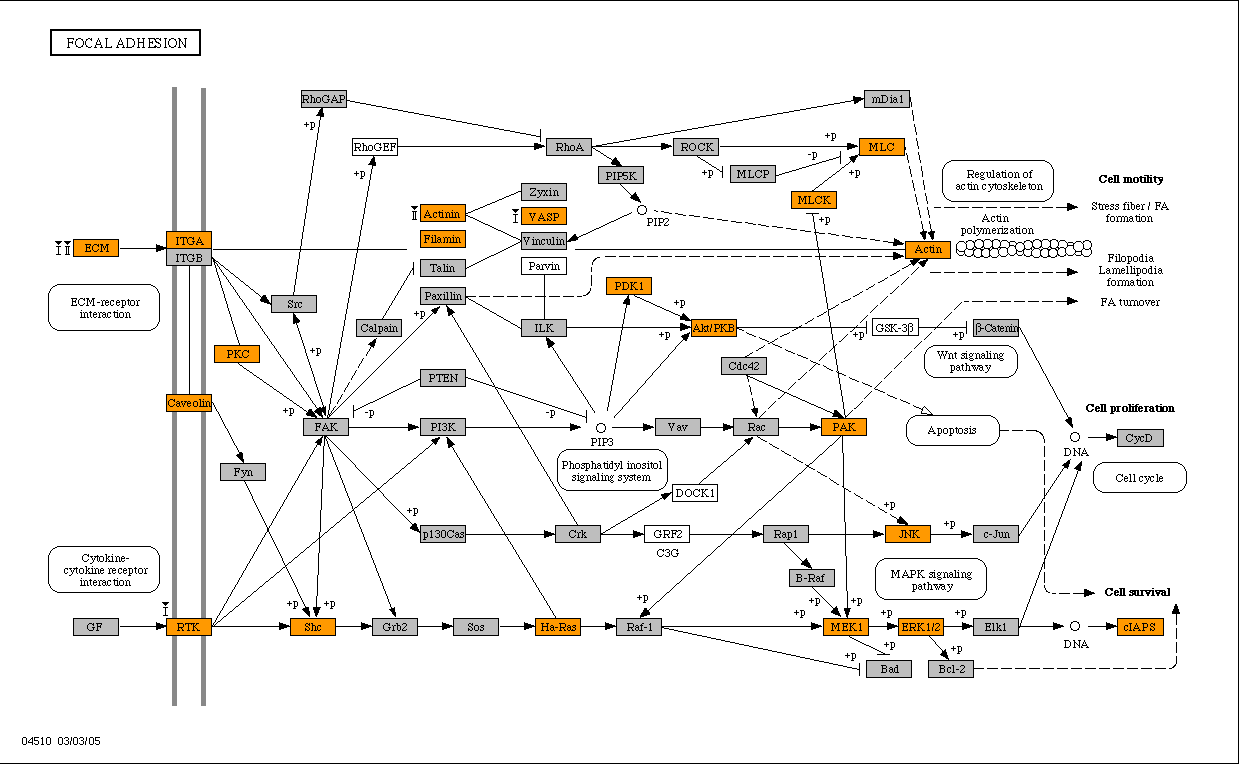
**Bimodal genes highlighted in KEGG focal adhesion diagram**. Nodes representing switch-like genes are colored orange; remaining genes on the MG-U74Av2 array are shown in grey, and white blocks designate genes not on the array. Genes identified as switching in skeletal muscle samples with diabetes type I and type II are labeled with "I" and "II", respectively, with arrows indicating the direction of change in the disease state (▲ is low-to-high; ▼ is high-to-low).

Interaction with the extracellular environment appeared to be a common theme when we tested our bimodal gene subsets for enrichment among KEGG pathways and GO terms. Our findings for enriched KEGG pathways and GO categories are summarized in Tables 3 and 4, respectively. The tables include enrichment scores, defined as the ratio of the observed number of genes over the expected number of genes from the subset of interest, and p-values for each entry calculated from a hypergeometric test [42] for the bimodal gene set against all unique genes on the MG-U74Av2 array. KEGG pathways that were enriched for bimodal genes include cell communication, ECM-receptor interaction, focal adhesion – all pathways that mediate cell communication with the extracellular environment (Table 3). Figures 2 and 3 identify the placement of bimodal genes (marked as orange) in ECM-receptor interaction and focal adhesion pathways, respectively. Structural proteins that are bound by integrin receptors – collage, laminin and fibronectin subunits – are largely encoded by bimodal genes, confirming the fact that the multiple signaling roles of integrins are coupled with the extracellular environment [43]. The focal adhesion pathway shown in Figure 3 illustrates bimodal genes that mediate cell communication at the interior of the cell including genes that encode proteins involved in phosphorylation (ERK1/2, JNK, MEK1, MLCK, PAK, and PDK1). Bimodal genes populate GO cellular component categories such as axons, basal lamina, basement membrane, cytoskeleton and extracellular matrix, and they are principally involved in the biological processes for ion transport, synaptic transmission, cytoskeletal organization and cell adhesion (Table 4). The abundance of genes with bimodal expression within the cell communication, focal adhesion, and ECM pathways suggests aspects of these activities are enabled and disabled at the transcript level. Additionally, KEGG pathways for sugar metabolism are enriched with bimodal genes, reminiscent of the switch-like regulation of lactose metabolism in bacteria.

**Table 3:** Enriched KEGG pathways for switch-like genes. Enrichment scores are the number of observed genes over the number of expected genes. P-values were computed using the hypergeometric distribution.

KEGG pathway	Genes observed	Genes expected	Ration of enrichment	P-values ≤ 0.01
Cell Communication	35	14.37	2.44	1.01E-07
ECM-receptor interaction	26	10.69	2.43	4.57E-06
Focal adhesion	42	24.23	1.73	1.47E-04
Fructose and mannose metabolism	13	5.68	2.29	2.18E-03
Glycolysis/Gluconeogenesis	16	8.19	1.95	4.61E-03
Long-term depression	17	9.36	1.82	8.07E-03
Tight junction	24	12.87	1.87	1.23E-03

**Table 4:** Enriched GO terms for switch-like genes. Terms are organized by cellular component (CC), biological process (BP), and molecular function (MF). Enrichment scores are the number of observed genes over the number of expected genes. P-values were computed using the hypergeometric distribution.

	GO term	Genes observed	Genes expected	Ration of enrichment	P-values ≤ 0.001
CC	axon	19	7.02	2.71	1.40E-05
	basal lamina	9	2.67	3.37	3.68E-04
	basement membrane	14	5.35	2.62	2.94E-04
	collagen	14	5.01	2.79	1.26E-04
	cytoskeleton	65	39.27	1.66	1.35E-05
	extracellular matrix	7	1.84	3.81	6.34E-04
	extracellular matrix (sensu Metazoa)	63	26.57	2.37	0.00E+00
	muscle myosin complex	6	1.17	5.13	1.30E-04
	postsynaptic membrane	18	7.85	2.29	3.20E-04
	sarcolemma	8	2.17	3.68	3.47E-04
	sarcoplasmic reticulum	6	1.34	4.49	4.44E-04
	synapse	24	9.52	2.52	5.03E-06
	synaptic vesicle	15	6.35	2.36	6.87E-04
	synaptosome	12	4.51	2.66	6.69E-04
	troponin complex	6	1.34	4.49	4.44E-04

BP	calcium ion transport	19	8.02	2.37	1.32E-04
	cell adhesion	88	50.79	1.73	4.41E-08
	cytoskeleton organization and biogenesis	29	14.54	1.99	1.09E-04
	ion transport	62	42.11	1.47	7.54E-04
	muscle contraction	24	8.86	2.71	1.03E-06
	muscle development	21	9.19	2.29	1.10E-04
	regulation of long-term neuronal synaptic plasticity	4	0.67	5.98	7.77E-04
	regulation of muscle contraction	14	3.01	4.65	1.97E-08
	striated muscle contraction	9	2.51	3.59	1.89E-04
	synaptic transmission	32	15.87	2.02	3.87E-05
	synaptogenesis	4	0.67	5.98	7.77E-04

MF	actin binding	41	24.39	1.68	3.61E-04
	calcium ion binding	113	78.70	1.44	2.03E-05
	creatine kinase activity	6	1.00	5.99	2.16E-05
	extracellular matrix structural constituent	23	9.69	2.37	2.57E-05
	extracellular matrix structural constituent conferring tensile strength	13	4.51	2.88	1.48E-04
	ion channel activity	44	25.40	1.73	1.05E-04
	kainate selective glutamate receptor activity	4	0.67	5.98	7.77E-04
	motor activity	30	13.03	2.30	3.33E-06
	protein binding	502	443.45	1.13	1.87E-04
	structural constituent of cytoskeleton	29	13.70	2.12	3.17E-05
	structural constituent of muscle	15	4.01	3.74	5.99E-07
	structural molecule activity	64	37.76	1.69	6.88E-06

### Altered modes of bimodal genes in diabetes

We identified bimodal genes that are expressed in an alternate mode within disease states for type I and type II diabetes. Comparisons of microarray samples for diabetes against samples for healthy tissue yielded nearly 200 genes with expression changes from "low" to "high" and "high" to "low" expression modes in skeletal muscle (Table 5). Changes were dominated by switching from "high" to "low" in skeletal muscle in both type I and type II diabetes. The bimodal genes with altered states in diabetes type I and II are enriched in pathways involved in communication and natural killer cell mediated cytotoxicity (Table 6). Additional file 2 provides a list of all bimodal genes with altered modes of expression in diabetes. The bimodal genes altered in diabetic skeletal muscle are mapped onto the ECM-receptor interaction and focal adhesion pathways, shown in Figures 2 and 3, respectively. As shown in these figures, collagen, fibronectin and tenascin are downregulated in diabetes type I and II transitioning from "high" to "low" expression whereas collagen receptor CD36 is switched from "low" to "high" expression in diabetes, perhaps as compensation for lower expression of extracellular matrix proteins. The list of bimodal genes association with diabetes may provide clues as to the changes that occur in gene regulation pathways as a result of the disease. Bimodal genes have also been implicated in congestive heart failure, Alzheimer's disease, arteriosclerosis, breast neoplasms, hypertension, myocardial infarction, obesity, and rheumatoid arthritis. Future studies are needed for a comprehensive portrayal of their roles in these various diseases.

**Table 5:** Switch-like genes identified in alternate modes of expression in diabetes. Quantities of bimodal genes that are in alternate modes between healthy tissue and disease tissue are shown for diabetes type I and II.

	Tissue type:	Adipose	Heart	Liver	Muscle	Pancreas	Peripheral Blood
Type I diabetes	"low" to "high"	---	13	---	37	16	9
	"high" to "low"	---	9	---	130	2	16

Type II diabetes	"low" to "high"	2	---	11	25	16	---
	"high" to "low"	3	---	14	142	3	---

**Table 6:** Enriched KEGG pathways for switch-like genes identified in alternate modes of expression in diabetes. Enriched KEGG pathways are shown for bimodal genes that switch states between healthy tissue and type I and II diabetes (T1 and T2, respectively). Enrichment scores are the number of observed genes over the number of expected genes. P-values were computed using the hypergeometric distribution.

	Tissue	KEGG pathway	Genes observed	Genes expected	Ration of enrichment	P ≤ 0.01
	Heart	---	---	---	---	---
T1	Muscle	Antigen processing and presentation	9	1.05	8.60	8.11E-07
		Cell communication	7	1.58	4.43	9.89E-04
		Cell adhesion molecules (CAMs)	8	1.73	4.63	3.19E-04
		ECM-receptor interaction	7	1.18	5.95	1.60E-04
		Focal adhesion	9	2.66	3.38	1.38E-03
		Leukocyte transendothelial migration	8	1.58	5.06	1.72E-04
		Natural killer cell mediated cytotoxicity	6	1.67	3.59	6.47E-03
		Type I diabetes mellitus	5	0.77	6.48	9.67E-04
	Pancreas	Benzoate degradation via hydroxylation	1	0.01	101.01	9.86E-03
		Leukocyte transendothelial migration	3	0.17	17.62	6.02E-04
		Tight junction	2	0.15	13.12	9.92E-03
	Peripheral blood	---	---	---	---	---

T2	Adipose	---	---	---	---	---
	Liver	PPAR signaling pathway	2	0.15	13.47	9.52E-03
	Muscle	Antigen processing and presentation	8	1.05	7.64	8.42E-06
		Cell communication	6	1.58	3.80	4.92E-03
		Cell adhesion molecules (CAMs)	8	1.73	4.63	3.19E-04
		ECM-receptor interaction	6	1.18	5.10	1.09E-03
		Leukocyte transendothelial migration	8	1.58	5.06	1.72E-04
		Natural killer cell mediated cytotoxicity	6	1.67	3.59	6.47E-03
		Type I diabetes mellitus	5	0.77	6.48	9.67E-04
	Pancreas	---	---	---	---	---

### Transcription factors and bimodal gene expression

Approximately 15% of transcription factors are bimodal. Comparison of our bimodal gene list with the transcription factor list obtained from the Transfac Professional Database [44,45] revealed 76 out of a total 525 transcription factors on the MG-U74Av2 array as bimodal (see Additional file 3). In turn, binding sites for these transcription factors have been identified for 91 genes with Entrez gene IDs, 79 of which were on the MG-U74Av2 array. Only 25 out of these 79 genes were bimodal, indicating that the set of bimodal transcription factors may not be solely responsible for their regulation. Nevertheless, genes that are regulated or co-regulated by bimodal transcription factors are enriched in some of the same KEGG pathways as bimodal genes (Table 7), including cell communication and ECM-receptor interaction. The GO categories for the genes co-regulated by switch-like transcription factors also intersect with GO categories of switch-like genes that are not transcription factors (Table 8). Additional file 3 shows that genes coding transcription factors that are involved in development such as the homeo box genes are switch-like. As the list of known transcription factors and their binding sites grow in the near future, more definitive relationships between bimodal genes and transcription factors are likely to emerge.

**Table 7:** Enriched KEGG pathways for genes regulated by switch-like transcription factors. Enrichment scores are the number of observed genes over the number of expected genes. P-values were computed using the hypergeometric distribution.

KEGG pathway	Genes observed	Genes expected	Ration of enrichment	P-values ≤ 0.01
Antigen processing and presentation	6	0.64	9.40	3.07E-05
Cell adhesion molecules (CAMs)	5	0.97	5.17	2.54E-03
Cell Communication	5	1.01	4.96	3.05E-03
Circadian rhythm	3	0.19	16.19	6.45E-04
ECM-receptor interaction	4	0.74	5.40	5.94E-03
Maturity onset diabetes of the young	3	0.43	6.94	8.55E-03
PPAR signaling pathway	5	0.82	6.07	1.22E-03
Type I diabetes mellitus	7	0.56	12.60	7.63E-07
Type II diabetes mellitus	4	0.66	6.07	3.86E-03

**Table 8:** Enriched GO terms for genes regulated by switch-like transcription factors. Terms are organized by cellular component (CC), biological process (BP), and molecular function (MF). Enrichment scores are the number of observed genes over the number of expected genes. P-values were computed using the hypergeometric distribution.

GO term		Genes observed	Genes expected	Ration of enrichment	P-values ≤ 0.001
CC	collagen type I	2	0.04	48.58	4.18E-04
	external side of plasma membrane	6	0.93	6.48	2.70E-04
	extracellular matrix (sensu Metazoa)	8	1.63	4.92	1.80E-04
	extracellular region	14	4.43	3.16	9.44E-05
	extracellular space	30	13.44	2.23	5.68E-06
	MHC class I protein complex	3	0.14	20.82	2.77E-04

BP	antigen processing and presentation of endogenous peptide antigen via MHC class I	2	0.04	48.58	4.18E-04
	antigen processing and presentation of peptide antigen via MHC class I	3	0.14	20.82	2.77E-04
	defense response	6	1.01	5.95	4.34E-04
	glucose transport	4	0.23	17.67	4.92E-05
	immune response	11	3.23	3.40	3.07E-04
	rhythmic process	3	0.19	16.19	6.45E-04
MF	extracellular matrix structural constituent	5	0.56	9.00	1.84E-04
	structural constituent of bone	2	0.04	48.58	4.18E-04

## Discussion

This article presents a comprehensive list of bimodal genes in the mouse genome. We used an automated statistical algorithm that is similar to the approach used in the detection of bimodality in blood glucose distribution [46,47] in order to identify bimodal, switch-like genes in a large-scale microarray database for murine tissue. Bimodal gene expression is either in a "high" or "low" expression mode, indicating switch-like regulation at the transcript level. Our automated analysis revealed over 15% of the genes in the mouse genome as bimodal (switch-like). These bimodal genes are enriched in cell communication pathways and are also enriched in such biological processes as cell adhesion, synaptic transmission, and ion transport. Moreover, bimodal genes associate with a large number of disease types including diabetes type I and II, hypertension, and cancer. Because a large portion of bimodal gene products are positioned in the extracellular region, the list we present in this study provides potential biomarker targets for early detection and accurate classification of complex diseases.

Although we have paid considerable attention to the statistics of identifying bimodal genes from the large-scale microarray data, our list of bimodal genes may change with time as microarray data obtained with the same Affymetrix system expands to include tissue types not considered in this study. Nevertheless, the list that we present in this article is stable under deletion of gene expression outliers from the data. Although, as discussed in the Background section, a number of genes from various species have been identified in the literature as bimodal or switch-like previously, to our knowledge, the list that we present (1519 genes) is yet the most comprehensive and contains important information on gene regulation in health and disease at the transcript level. Although the list annotates bimodal genes for the murine genome, their orthologs presented for the human genome provide a core candidate list for the bimodal genes in the human genome. Our automated method for annotating bimodal genes will yield a comprehensive list for the human genome with the availability of a comprehensive set of standardized microarray data for large numbers of well controlled tissue samples.

Recent literature points to examples of bimodal genes involved in feedback and feedforward motifs in gene regulation networks [48-50]. Bimodal gene expression associated with switch-like regulation was shown to be a direct consequence of DNA methylation at cis-regulatory sequences at least in the case of E-coli metabolic gene circuitry [51]. This observation is consistent with our finding that only a small number of transcription factors are bimodal and those transcription factors in turn only regulate a small portion of the remaining bimodal genes.

Our study indicates that in a number of complex diseases such as diabetes type I and II, the stable inheritance of the normal mode of expression in bimodal genes is compromised. For example, bimodal genes coding for collagen subunits are "low" rather than "high" in skeletal muscle for diabetes type I and II relative to healthy samples. In addition, type II diabetes has the fibronectin subunit gene "low" rather than "high" in the same tissue. Perhaps, in compensation, collagen receptor CD36 becomes highly expressed in both diabetes types. Our comprehensive list of bimodal genes in the mouse will be useful in identifying disease-phenotypic alterations in gene regulation in diseases such as cancer, hypertension and diabetes.

For the interest of assessing the diagnostic potential of switch-like genes as biomarkers, we compared our switch-like gene list with previously published lists of serum proteins and disease genes. Mouse orthologs were obtained for serum proteins identified in the HuPO PPP, including the 3020 two-plus peptide list and 889 high-confidence lists [52,53]. We found that nearly a quarter of the high-confidence plasma proteins were bimodal. Although these results may change as more accurate proteomic measurements are available, it indicates the potential of switch-like genes as biomarkers for the classification of disease subtypes.

We compared our list of bimodal genes with disease gene sets for mouse obtained from the RGD Disease Portal [54]. On the average, we identified that bimodal genes account for 15% of the genes within disease gene lists for congestive heart failure, Alzheimer's disease, arteriosclerosis, breast neoplasms, cerebrovascular accident, hypertension, myocardial infarction, obesity, rheumatoid arthritis, and diabetes mellitus types I and II. Among these bimodal genes, 30% were serum protein encoding genes, suggesting their potential to serve as biomarkers.

## Conclusion

This research identified a large set of mouse genes as switch-like by assembling and analyzing a large collection of microarray data encompassing diverse tissue types. Genes with bimodal, switch-like control were shown to be enriched within the cell communication pathways and the extracellular environment. The modes of expression for a large majority of such genes were tissue-selective. Moreover, a significant number of these switch-like genes switched between modes of expression in diabetic compared to healthy samples in a number of tissue types. These findings comprise an important first step in identifying altered states of gene switches in complex diseases such as hypertension, obesity and cancer.

Bimodal expression implicates strong regulation at the transcript level. Switch-like regulation can influence protein activity in cases where protein abundance parallels transcript level, as is observed with proteins such as cytokines [55,56]. Bimodal gene expression provides a means for the cell to enable and disable pathway functions at the transcript level. Genes with bimodal, switch-like control are involved in communication pathways that play crucial roles in determining cell phenotype through interaction with the extracellular environment in health and disease. Because their expression is tightly regulated at the transcription level, they comprise a candidate set of biomarkers for a large number of disease states.

## Methods

### Data selection

Murine gene expression datasets (Table 1) were obtained from both the Gene Expression Omnibus (GEO,) [57-59] and ArrayExpress [60,61] and limited to those providing Affymetrix GeneChip MG-U74Av2 data in CEL file format, because datasets are both current and abundant for this platform. The resulting dataset contains samples from nineteen generalized tissue types. While only one sample was obtained for stomach tissue, this does not seem to impact the detection of switch-like or tissue-selective genes identified within this tissue. Moreover, stomach tissue clusters with other digestive tissues based on the intersection of tissue-selective gene sets, as presented in the results section.

### Microarray normalization and annotation

Robust Multichip Average (RMA) [62,63] expression values were computed from these CEL files, using RMAExpress software version 0.5 Release [64] with default settings, to produce a data table with genes as rows and samples as columns. All CEL files from datasets listed in Table 1 were used for normalization, but the data was limited to healthy subjects, excluding knockout and disease phenotypes, for subsequent steps in the analysis. Annotation for Entrez Gene ID, gene symbol, and KEGG pathway was retrieved March 15th, 2007 using Webgestalt (web-based gene set analysis toolkit) [42]. GO annotation, as well as missing values for Entrez Gene ID and gene symbol, was supplemented from the MG-U74Av2 annotation dated 3/8/2007, obtained directly from the Affymetrix website [65] on March 15th, 2007. The data was then imported to Matlab R2006b (The Mathworks Inc., Natick, MA, USA), where all subsequent procedures were implemented.

### Disease markers, serum proteins, and transcription factor annotation

Disease gene sets for mouse were obtained from the Rat Genome Database (RGD) Disease Portal [54]. Mouse orthologs were obtained for serum protein lists available from the Human Proteome Organization (HuPO) Plasma Proteome Project (PPP) [52,53]. Entries in the PPP list were converted from International Protein Index (IPI) to human Entrez Gene ID using the IPI database for HUMAN, version 3.28, released 20 Apr 2007 [66,67]. Mouse orthologs were obtained from this list using Webgestalt. The Transfac Professional Database version 11.1 [44,45] was used to identify genes as either transcription factor coding genes or transcription factor targets. Gene entries, including those encoding for transcription factors, were obtained from Transfac Gene and limited to those with Entrez Gene IDs represented on the MG-U74Av2 array.

### Identification of bimodal genes from estimated parameters for two-component mixtures

Bimodal genes in the murine microarray data (Table 1) were identified using a statistical method applied to bimodality in glucose distribution [46,47]. Briefly, we tested the hypothesis H_1 _that gene expression distribution follows a two-component (bimodal) mixture against the hypothesis H_0 _of a single normal distribution, adjusted for skewness. For this purpose, we used the box-cox transformation implemented in Matlab to eliminate skewness in RMA-adjusted gene expression histograms for each gene in the microarray database [68]. Then we used the expectation maximization (EM) algorithm [69] implemented in Matlab to determine the parameters for the best-fit two-component normal mixture for each gene in the database. The two-component normal mixture is used to represent a bimodal distribution, where the parameters μ_1 _and μ_2 _are the component means, σ_1 _and σ_2 _are the component standard deviations and π_1 _and π_2 _represent the proportion of data within each component (note that π_2 _= 1 - π_1_). The corresponding parameters for single normal distribution were calculated from the sample mean and standard deviation for each gene. The log likelihood ratio test statistic -2logλ was computed for the two-component mixture hypothesis H_1 _versus the null hypothesis H_0 _of a single component as described in [46,47]. We estimated the p-values for two-component mixtures by evaluating the chi-square distribution with six degrees of freedom (DF) at the values of -2logλ. The asymptotic null distribution for the -2logλ statistic is typically represented by a chi-square distribution with DF equal to the difference in the number of parameters between the null and alternative hypotheses. However, regularity conditions for -2logλ do not hold in the case of mixture models, and simulation has shown that six DF is a more accurate representation for the asymptotic null distribution when testing the alternative hypothesis of two components with unequal variance [70]. The choice of six degrees of freedom for testing two components versus a single normal provides conservative p-values and was previously used in identifying bimodality in blood glucose levels among population subsets [47].

Candidates for bimodal "switch-like" genes were selected as those with p-values no more than 0.001, which produced a subset of 2166 out of 9091 unique genes on the MG-U74Av2 array. The table in Additional file 1 lists those candidate genes with p < 0.01 in order to identify additional genes that might also be considered bimodal with additional biological context, though only genes with p < 0.001 were included in our analysis to keep the false discovery rate low for the 9091 genes under consideration. In order to investigate the effect of outliers on the prediction of bimodality from gene expression data, we ran the EM procedure again within the set of bimodal candidate genes leaving out the three highest expression values for each gene. Five genes came out of the candidate list and are highlighted with an asterisk (*) in Additional file 1 though they were not excluded from our final list.

**Figure 4:**
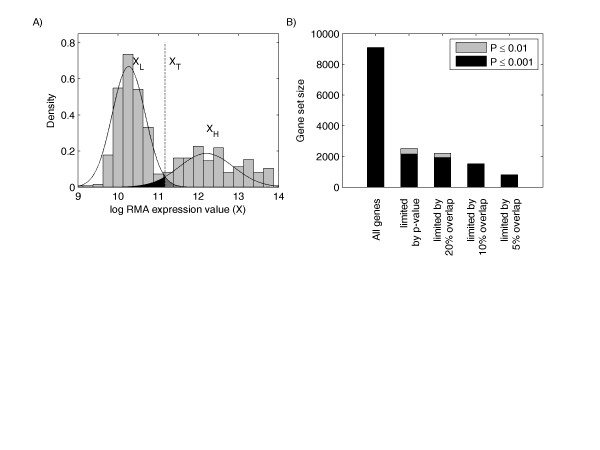
**Identification of switch-like genes with bimodal gene expression**. A) The histogram and normal mixture probability density function (pdf) for a bimodal candidate gene. B) Bar graph representing gene set sizes during switch-like gene candidate selection process.

This subset of genes was further reduced by the imposing the requirement that the standardized area of intersection A (indicating type I and type II error for the estimated bimodal distribution divided by the total area) was less than 0.10. This is clarified in Figure 4A, where the dark grey region under the normal curves represents type I error and the solid black region under the normal curves represents type II error. The rationale for this step is that in order for switching to play a functional role within the cell, there must be minimal overlap between the two mixture components. This criterion reduced the number of candidate switch-like genes from 2166 to 1458. In the resulting gene list, the standardized distance between components, D = (μ_2 _- μ_1_)/min(σ_1_, σ_2_), was at least 2.5 for this remaining subset of switch-like gene candidates, confirming the statistical power of our analysis [71]. Figure 4B illustrates the reduction in switch-like gene candidates based on several cutoff values for the type I and II error rate.

### Assigning expression values to high/low modes for switch-like genes

The switching threshold for each gene was defined at the intersection of the density curves for the two components of the mixture. This switching threshold is mapped back onto the log RMA expression axis (labeled as *X*_*T *_in Figure 4A) with a reverse box-cox transformation. A gene expression sample greater than *X*_*T *_*for *that gene was classified as having high expression, while a sample less than *X*_*T *_*was *classified has having low expression. Standardized area of intersection A was restricted to less than 0.1 in order to keep classification error to a minimum in the assignment of "high" and "low" states to switch-like genes.

### Developing a dendrogram for tissue similarity using the concept of tissue selectivity

Nineteen tissue types for which we have extensive gene expression profiling have been clustered in dendrogram using a coexpression matrix. Elements of the coexpression matrix were selected from the larger gene set with the restriction that a gene in the subset must be expressed in the high mode for the majority of the samples in at least one tissue. For this purpose, the gene expression values for switch-like genes were converted to binary values corresponding to the high and low modes of two-component distribution. Gene expression within a single tissue type was modeled as a Bernoulli process (binomial distribution) with equal probabilities of high and low. Based on this model, a gene that is not selectively expressed within a given tissue type should be evenly distributed between the high and low modes. A gene that is selectively expressed within a given tissue type would show a significant bias for the high mode and low corresponding p-value. We established p-values for a gene to be selectively expressed within each tissue type from the binomial distribution, where the number of trials equals the number of samples for that tissue type and the number of successes equals the number of samples with values in the high expression component. Conversely, p-values for a gene to be selectively repressed within each tissue type were established based on the number of samples with values in the low expression component. Tissue-selective genes were selected as those with p < 0.01 for at least one of the nineteen general tissue types.

### Functional enrichment analysis

KEGG pathway and GO annotations were used to compute functional enrichment scores for all switch-like genes. Functional enrichment analysis was performed in Matlab by calculating the ratio of genes belonging to a functional category within a gene set of interest against the total number of genes belonging to that functional category within the set of genes on the MG-U74Av2 array. Enrichment p-values were computed from a hypergeometric distribution. The p-value cutoffs were selected at 0.01 for KEGG pathways and 0.001 for GO terms, to reduce the false discovery rate. The set of candidate bimodal genes contained 153 unique KEGG pathways and 321 unique GO cellular component terms, for which an expected 1.5 and 0.3 of the terms may appear significant by chance at these p-value cutoffs, respectively.

### Comparisons of health and disease states

Additional MG-U74Av2 samples were used to identify alternate switching modes of switch-like genes in diabetes type I and II. These samples are listed in Table 2 and represent adipose, heart, liver, skeletal muscle, pancreas, and peripheral blood. Bimodal genes were identified as altered in disease for a single tissue by again modeling the samples as a binomial distribution. Genes were identified as switching in the disease state when healthy samples have a significant p-value (less that 0.01) in one mode while disease samples have a significant p-value (less than 0.01) for the opposite mode.

## Authors' contributions

AE and AT worked together on this project. AE implemented the algorithms, performed the computations and provided a first draft for the manuscript. Both authors read and approved the final version of the manuscript.

## Supplementary Material

Additional file 1 **Bimodal gene list**. This table provides a comprehensive list of bimodal genes for the mouse genome including parameters used in the identification of bimodal gene expression. Click here for file

Additional file 2 **Bimodal genes with altered modes of expression in diabetes**. This table identifies bimodal genes that switch between high and low modes of expression in type I and II diabetes vs. normal within various tissue types. Click here for file

Additional file 3 **Transcription factor genes with bimodal expression**. This table lists bimodal genes identified as transcription factor coding in the Transfac Professional Database. Click here for file
